# Scientific Challenges in Performing Life-Cycle Assessment in the Food Supply Chain

**DOI:** 10.3390/foods8080301

**Published:** 2019-08-01

**Authors:** Ilija Djekic, Milica Pojić, Alberto Tonda, Predrag Putnik, Danijela Bursać Kovačević, Anet Režek-Jambrak, Igor Tomasevic

**Affiliations:** 1 Faculty of Agriculture, University of Belgrade, Nemanjina 6, 11080 Zemun, Serbia; 2 Institute of Food Technology, University of Novi Sad, Bulevar cara Lazara 1, 21000 Novi Sad, Serbia; 3 Institut National de la Recherche Agronomique (INRA), 1 Avenue Lucien Brétignères, 78850 Thiverval-Grignon, France; 4 Department of Food Engineering, Faculty of Food Technology and Biotechnology, University of Zagreb, 10 000 Zagreb, Croatia

**Keywords:** life-cycle assessment, food supply chain, system boundaries, functional units, sensitivity

## Abstract

This paper gives an overview of scientific challenges that occur when performing life-cycle assessment (LCA) in the food supply chain. In order to evaluate these risks, the Failure Mode and Effect Analysis tool has been used. Challenges related to setting the goal and scope of LCA revealed four hot spots: system boundaries of LCA; used functional units; type and quality of data categories, and main assumptions and limitations of the study. Within the inventory analysis, challenging issues are associated with allocation of material and energy flows and waste streams released to the environment. Impact assessment brings uncertainties in choosing appropriate environmental impacts. Finally, in order to interpret results, a scientifically sound sensitivity analysis should be performed to check how stable calculations and results are. Identified challenges pave the way for improving LCA of food supply chains in order to enable comparison of results.

## 1. Introduction

Food systems, their diversity and interaction with the environment have been hot research topics for many years [1]. Recent European consumption studies acknowledged the responsibility of food production for almost half of all known and measured environmental impacts [2,3]. This triggered the development of environmental requirements that directly or indirectly cover various ecological impacts of food production and all of its (sub)processes [4]. As a result, various strategies within food supply chains have been modeled, covering stakeholders from farms to retailers and households [1] that are focused on sustainable and environmentally sound improvements [5,6,7].

For the purpose of analyzing all stages of agricultural and food production, life-cycle assessment (LCA) is very often used to compute environmental impacts [8]. It is considered as a powerful tool for evaluating the impact of human and food related industrial activities on the environment [9]. As a very data-intensive methodology where a life-cycle may cover up to thousands of human/industrial activities, it is important to understand and documented them [10]. As outlined in the ISO 14040 international standard, a LCA consists of four main phases: (i) defining the goal and scope of a LCA; (ii) analysis of the inventory necessary for carrying out the study; (iii) calculation and assessment of environmental impacts; and (iv) interpretation of the results [11].

Depending on the type of food, first challenge within the ‘goal and scope definition’ phase is setting adequate system boundaries. To determine environmental impacts that mostly occur throughout the food supply chain, a ‘farm-to-fork’ perspective is crucial to map all of food process(es) that are joined with the scope and boundaries of the food system [1]. LCA of a food product should clearly specify the function of the product, rather than to focus on the food product itself.

The measure of (environmental) performance, which the system delivers, is called a functional unit (FU). The FU provides a reasonable point of reference when comparing various food products and its proper selection is of a paramount importance. Especially, since different FUs can pave the way to a different outcomes for the same food system [12]. Great majority of the LCA food studies employs physical units for FUs, such as mass of food (kg/t), volume of food (L/m^3^), or area associated with farms (ha) [13].

The type and quality of data categories included in the LCA study represent the hot points that need to be identified. Although, collecting data is crucial it is very challenging, since various types of uncertainties are associated with imprecise numeric values [13]. Data for each process unit consists of physical inputs (energy and raw materials), products coupled with all co-products and outputs in terms of waste disposal, emissions to air or discharge of various pollutants to the soil and water [11,12].

Life-cycle inventory (LCI) is recognized as a time consuming step associated with collection of data from the subsystems, as well as aggregation and validation of the data [1]. During this phase, all input-flows related to materials and energy together with all waste-streams associated with environmental pollution throughout the investigated life-cycle of the food system are identified and quantified. The inventory phase has four sub-stages: (a) constructing a process flow chart; (b) collecting the data; (c) relating the data to a chosen FU (allocation); and (d) developing an overall energy and material balance. The last stage should cover all inputs and outputs from the entire life-cycle while everything is represented as an inventory table. Allocation is a process that fulfils two (or more) functions or gives two (or more) usable outputs. It is necessary to determine which part of the total emission and material/energy consumption should be attributed to each specific product.

Impact assessment brings uncertainties in terms of choosing appropriate environmental performance metrics, since there are broadly recognized environmental potentials. When environmental assessment are using endpoints to convey information on the impacts, they deploy potentials to three main damages, namely: human health, ecosystems and resources [14]. However, the choice of appropriate categories has subjective nature.

Herva, et al. [15] recognized three major footprints pertaining to ecology, water, and carbon footprint. Ecological footprint evaluates environmental consequences of human pressures on the biosphere and ecosystems and measures human appropriation of planet’s regenerative capacity [16]. In food supply chains, it should be tracked down how much of Earth’s regenerative capacity is needed, as compared to how much of regenerative capacity is available, as ecological assets (e.g. crop, grazing, forestation, fishery, carbon built-up and uptake by the lands) [17]. Broadly speaking, known potentials represent resource depletion associated with climate change. This is focused on sources of water and energy, global warming and ozone depletion, additional to toxicity from related stressors, such as eutrophication and acidification. Carbon footprint is the most accepted benchmark for environmental impacts in the meat and dairy chains [18,19].

Environmental impacts that occur within the food supply chain may have an influence on humans. Human potential for toxicity reflects the potential for harm (exposure and/or accumulation) of a substances, residues, effluents or emissions on human health [20]. It is based on the inherent toxicity of a compound and its potential dosage [21], and can be a liquid effluent, a solid residue or a gaseous emission [20]. In LCA assessments, when this potential is calculated, it considers fate, exposure, and effects of toxic substances for an infinite time horizon and is expressed as 1,4-dichlorobenzene equivalents/kg of emission [22].

Finally, in order to interpret results, a scientifically sound sensitivity analysis should be performed to check how stable all calculations and results are. This analysis is carried out to differentiate between the influence and the importance of an input data on the output [13]. It also helps in deploying mitigation strategies to overcome this issue [23,24].

The main objective of this study was to evaluate challenges when performing a LCA in the food supply chain using Failure Mode and Effect Analysis to weigh potential risks that may occur in all stages of LCA assessments.

## 2. Materials and Methods

Ranking of risks associated with LCA in food supply chains was performed by professionals with expertise in life-cycle assessment and modelling from the Universities of Belgrade, University of Novi Sad (Serbia), University of Zagreb (Croatia), and INRA (France). The team decided to use Failure Mode and Effects Analysis (FMEA) as an engineering analytical tool in ranking the risks [25] for LCA of a food product. This technique enables identification of possible failure modes and their causes, but also helps in investigating the effects of the failures on system performance [26]. It is common to have a team of experts to perform a comprehensive FMEA [27], joint with the use of information/knowledge and associated with similar items or problems [28]. Basic FMEA protocol comprises of compiling an inventory of possible failure modes and evaluating the risks associated with them [29]. In our case, we identified a list of potential nonconformities related to performing a LCA study. FMEA risk called the “risk priority number (RPN)” is calculated by multiplying three factors [26]:

(1) RPN=S × O × D

where: (S) represents severity of the failure in performing a LCA; (O) indicates occurrence associated with the probability for a specific failure; and (D) stands for difficulty to detect that failure. Table 1 outlines weighting factors of the three factors.

Since there is a limited number of papers applying FMEA in food industry focused on environmental issues in food systems, to define these factors current study employed values from previous reports on GMO and food safety issues [28] with evaluating hygiene design risks in novel technologies [30].

Delphi method was applied to encourage experts from our team to determine the weight factors and to calculate the final risk. In order to achieve consensus, this method was used to elicit experts’ knowledge [31]. With its flexible approach as an iterative multistage process, it was designed to transform opinion(s) into consensus reached by a group of experts [32].

A total of ten experts participated in the session, and they confirmed that all important nonconformities that may occur while performing LCA in a food supply chain have been included. Before the session, a brief guideline for joint-reporting with empty FMEA forms were distributed to the experts, where they had one hour to weight all factors for the pre-defined nonconformities. There were no holdouts for any of the nonconformities in the first round. After a short discussion, in the second round consensus for each weighting factor was reached and there were no opposed and/or conflicting opinions for the final RPN score.

## 3. Results

### 3.1. Scope and Goal

Within the ‘scope and goal’ stage (Table 2), the highest risks were associated with the quality of data used in LCA and limitations of the study. Depending on data sources, they were classified as *primary data*, coming from direct sources, or *secondary data*, originated from supporting calculations. Primary sources are crucial where type and scale of data can vary from local to regional or global levels [33,34]. Data sources cover different scales from smaller samples of food producers [35] to a large business units, and even the entire industrial food sectors [36].

Time span plays an important role, as the data from the subsystems might not come from the same year/period, which brings the next important item—a consistency of data through entire life-cycle. For example, inconsistent functional unit risk scored 24 (Table 2). Following the data collection, it is necessary to validate the data and relate them to the right processes/products/FUs [11]. Assumptions and limitations of LCA study lead to the next group of issues in the first phase such as: (a) collection of data and calculation methods (on-site visits or identification of unit processes); (b) assumptions based on great variability and imprecise data; (c) limitations from (excluded) system boundaries (i.e., waste management scenario that includes only landfill, with no other waste approaches); and (d) limitations from (excluded) environmental impacts.

The type of a food-related company in the food supply chain influences the choice of functional units. In the case of meat production, common FUs are: one kg of livestock; one kg of carcass and/or one kg of meat or meat product [12]. In the poultry chain, FU can be expressed as one kg of live poultry linked to farms; one kg of carcass weight/packaged broiler chicken associated with poultry abattoirs; or one kg of chicken product related to selling points [37]. On the other hand, in the dairy sector common FUs are: 1 L of raw milk or 1 kg of dairy product [19]. However, since the main function of food is to feed the population, other FUs related to nutritional values may also be deployed such as: energy (KJ/cal); protein/digestible protein (g); total fat/saturated fat/trans-fat (g); carbohydrates/sugars (g) etc.

Usual data collection methods consist of creating a questionnaires/checklists and communicating it in advance with companies in the food supply chain. That way questionnaire is administered directly during the visit to such organizations. However, during the company visits, some additional activities may occur, such as various measurements, further estimations, and re-calculation. This is even more pronounced when examining small companies.

Setting up inadequate system boundaries contains certain risks for all stages of analysis that are not included. This is very important step, since the main technique applied in all LCAs is modelling the life-cycle. A ‘cradle-to-grave’ context consists of identifying and understanding all subsequent processes involved in food production/processing, with transportation, use, and disposal of foods. Here, the standard ISO 14040 identifies three main stages: (i) cradle-to-gate; (ii) gate-to-gate; (iii) gate-to-grave [11]. However, in many LCAs employed on food products, the ‘cradle-to-gate’ may be transformed to ‘cradle-to-market’, involving activities from farms to distribution and sales, or ‘cradle-to-use,’ covering the consumption phase [1].

Finally, for defining system boundaries, it is important to analyze the entire food supply chain in terms of subsystems. As an example, some authors recognized five subsystems in the meat supply chain as follows: (i) farms covering activities with livestock; (ii) facilities for slaughtering animals; (iii) meat and meat product manufacturers; (iv) meat selling points (butcher shops, retailers, and other types of shopping stores); and (v) meat and meat product consumers [12,38]. The dairy supply chain usually identifies four subsystems: (i) dairy farms; (ii) dairy plants; (iii) dairy products selling points; and (iv) dairy product consumers [19]. For both, meat and dairy chains, exclusion of feed production for livestock may bring additional inconsistencies when interpreting the results. This is also a vital for defining system boundaries in plant production in terms of duration of plant life-cycles [39,40].

### 3.2. Inventory Analysis

FMEA of this stage revealed one of the two highest risks designated to problems related to imprecise allocation. The severity was ranked ‘5’ since failures are associated with the final results throughout the entire life-cycle. Occurrence was ranked ‘2,’ since all calculated errors were referred to bad inputs, while detection was ranked ‘4,’ since failures associated with the final results were usually detected during the interpretation phase. Depending on the approach, typical allocation methods were physical allocation (based on mass/volume of food), economic allocation (calculated by prices or specific price-relations of food or even mass/volume weighted by prices), and system expansions [41].

Within inventory analysis, the main problem that occurs is how to divide emissions and material consumption between several product and processes. It is the ‘problem within the problem’ type of situation, as the inventory analysis is associated with calculating input flows (material and energy) and output flows (waste/discharge streams) in the entire life-cycle assessment of the food system. It may result with exclusion of some material and energy flows (e.g. primary/converted energy inputs from nature/ technosphere). Considering that sometimes may be difficult to collect all the inventory data for the LCA of a food product, it is common to use appropriate databases. Wernet, Bauer, Steubing, Reinhard, Moreno-Ruiz and Weidema [10] stated that inventory databases are dominant LCA constituents highlighting significance of unit-process data quality.

### 3.3. Impact Assessment

The final result of the LCA is a list of potential environmental impacts which leads to the second highest risk. Usually this list of scores, one for each category, determines the environmental profile of a food product or a food service. Depending on the methodology and software used, up to 30 different impacts may be calculated here. In food industry, typical impacts cover climate change (global warming potential), depletion of resources (energy/water), and land/water stressors such as acidification and eutrophication [42].

### 3.4. Interpretation of the Results

Sources of uncertainty in a food LCA are quality of data, e.g. subjective choice of system boundaries, allocation rules, functional units, and environmental impacts (Table 2). In order to analyze results between different LCA studies, it is required to have them comparable in terms of same FUs and levels of uncertainty [43]. Input parameters cause uncertainties as a result of natural variability or epistemic uncertainty [44]. Epistemic uncertainties are mainly unknown within the LCA and the only way to reduce them is to increase the knowledge about the system where analysis of the (cause) uncertainty can improve understanding or robustness of the results [45]. An uncertainty analysis may be employed to evaluate the robustness of the results in contrast to a reasonable range of variations associated with different LCI parameters [46]. According to the FMEA this issue was ranked as the highest risk.

Finally, it is common to carry out a sensitivity analysis and rate whether or not the input data and methodological choices influenced the final results of LCA. This activity was also rated with a high score. Lewandowska, Foltynowicz and Podlesny [43] were among the first to point out this issue and to develop sensitivity indicators (from highly sensitive to highly insensitive) for each of the influencers in a sensitivity analysis. One of the latest studies developed five different methods for the inventory stage for performing sensitivity analysis in the LCA (i.e. squared standardized regression coefficient, squared Spearman correlation coefficient, key issue analysis, Sobol’ indices, and random balance design) [45].

## 4. Conclusions

A FMEA-based approach for evaluating risks in performing LCA in food data can provide guidance to food and environmental scientists, database managers and life-cycle assessors to concentrate efforts to the most influential hotspots. Our results suggested that focusing on the quality of data and allocation methods, together with choosing appropriate environmental impacts, has tendency to improve current practices in performing LCA throughout entire food supply chain. In this capacity, promotion of the food supply chain, not only at a farm level, should receive benefits in the future. Finally, clear specification of functional units for specific food products will enable precise comparisons of the same type of foods and direct future environmental improvements.

## Figures and Tables

**Table 1:** Severity, Occurrence and Detection rating scale

**Severity**		
**Rank**	**Consequence**	**Description**
1	None	No failure(s)
2	Minor	Failure(s) associated with results for one inventory
3	Low	Failure(s) associated with results within one subsystem
4	Major	Failure(s) associated with results within more than one subsystem
5	Severe	Failure(s) associated with results throughout the entire life-cycle
**Occurrence**		
**Rank**	**Probability**	**Description**
1	Very unlikely	Minimal probability of occurrence of failure(s) as a result of force majeure
2	Unlikely	Occurrence of failure(s) only as a result of misuse of software
3	Possible	Occurrence of failure(s) only as a result of errors in man-made calculations/estimations
4	High probability	Occurrence of failure(s) will occur only for certain type of products
5	Certain	Occurrence of failure(s) in each subsystem of the entire life-cycle
**Detection**		
**Rank**	**Criteria**	**Description**
1	Very high	Failure(s) associated with results is easily detected
2	High	Failure(s) associated with results is detected during inventory phase
3	Low	Failure(s) associated with results is detected during impact assessment phase
4	Remote	Failure(s) associated with results is detected during interpretation phase
5	Never	No possibility of identifying failure(s) associated with results of LCA

**Table 2:** Failure Mode and Effect Analysis of LCA of food.

No	Stage	Non-Conformity	What Might Occur?	Potential Failure Effect?	Severity (S)	Occurrence (O)	Detection (D)	Risk
1	Scope and goal	Inadequate system boundaries	LCA does not include all product stages	Results will show only partial life-cycle of the food product	5	3	1	15
2	Scope and goal	Inconsistent functional units	Calculation and interpretation of the results in wrong functional units	No possibility of comparing results throughout the food supply chain and benchmark with other	4	3	2	24
3	Scope and goal	Inappropriate data collection method	Inadequate collection methods (measurement, estimation, combination, re-calculation, …)	Wrong/un-useful data collected	3	3	2	18
4	Scope and goal	Low level of data quality	Primary/secondary sources, time related dimension (data for specific time periods), consistency of data quality in the entire life-cycle	Bad results during validation of data and when relating the data to processes/products/FUs	3	3	4	36
5	Scope and goal	Wrong limitations	Exclusion of important data/stages/system boundaries/environmental impacts	Results will show only partial life-cycle of the food product	3	4	3	36
6	Inventory analysis	Material and energy flows	All material/energy flows (primary/converted energy, inputs from nature/from technosphere) not included	Results will show only partial life-cycle of the food product	3	3	3	27
7	Inventory analysis	Waste streams	Incorrect calculations of outputs to nature/technosphere	Results will show only partial life-cycle of the food product	3	3	3	27
8	Inventory analysis	Imprecise allocation	Incorrect allocation of the total emissions and material consumption attributed to each specific product	Results will show only partial life-cycle of the food product	5	2	4	40
9	Impact assessment	Lack of an accepted official list of environmental impacts	Subjective choice of environmental impacts	Wrong/un-useful environmental impacts calculated	5	2	4	40
10	Interpretation of results	Uncertainty	Quality of data, subjective choice system boundaries, allocation rules, functional units, environmental impacts	Results will show only partial life-cycle of the food product	3	3	4	36
11	Interpretation of results	High sensitivity	Sensitivity analysis shows that the input data and methodological choices influence the results of a LCA too much	Results will show only partial life-cycle of the food product	4	2	4	32
